# Update on Systemic Therapies for Metastatic/Unresectable Pheochromocytomas and Paragangliomas and Future Directions

**DOI:** 10.3390/cancers17223702

**Published:** 2025-11-19

**Authors:** Imani Ghosh, Olivia Benson, Jorge H. Hernandez-Felix, Frank I. Lin, Karel Pacak, Jaydira del Rivero

**Affiliations:** 1 Developmental Therapeutics Clinic, National Cancer Institute (NCI), National Institutes of Health, Bethesda, MD 20892, USA; imanighosh7@gmail.com (I.G.); olivet9638@gmail.com (O.B.); jorge.hernandezfelix@nih.gov (J.H.H.-F.); 2 Molecular Imaging Branch, National Cancer Institute (NCI), National Institutes of Health, Bethesda, MD 20892, USA; frank.lin2@nih.gov; 3 Center for Adrenal Endocrine Tumors, AKESO, 155 00 Prague, Czech Republic; karel@mail.nih.gov; 4 Eunice Kennedy Shriver, National Institute of Child Health and Human Development, National Institutes of Health, Bethesda, MD 20892, USA

**Keywords:** pheochromocytoma, paraganglioma, tyrosine kinase inhibitor, HIF-2α inhibitor, radiopharmaceuticals, immunotherapy, chemotherapy

## Abstract

Metastatic or unresectable pheochromocytomas and paragangliomas (PPGLs) remain rare but clinically challenging neuroendocrine neoplasms with limited curative options. Traditionally managed with surgery, radionuclide therapy, or cytotoxic chemotherapy, systemic treatments have historically achieved disease stabilization, rather than durable remissions. In recent years, however, the therapeutic landscape has evolved substantially. Radiopharmaceuticals such as ^131^I-MIBG and ^177^Lu-DOTATATE continue to play a pivotal role, achieving disease control in many patients. Cytotoxic regimens, particularly temozolomide, remain relevant, with some studies suggesting that SDHB-mutated PPGLs demonstrate a heightened sensitivity associated with MGMT promoter hypermethylation and reduced MGMT expression. Targeted agents are increasingly important: multi-kinase inhibitors such as sunitinib, anlotinib, and cabozantinib have shown meaningful activity. The landmark approval of belzutifan, a HIF-2α inhibitor, in 2025 represents the first oral targeted therapy for advanced/metastatic PPGL, which is particularly relevant for pseudohypoxic Cluster 1 tumors. Immunotherapy has yielded modest responses with checkpoint inhibitor monotherapy, but ongoing studies of dual checkpoint blockade and TKI–ICI combinations hold promise. Novel approaches, including PARP inhibition, metabolic targeting strategies, and cancer vaccines, are under investigation, especially for aggressive SDHB-related disease. Optimal sequencing of these therapies is emerging as a central challenge, with treatment strategies increasingly tailored to molecular genotype, tumor behavior, and functional imaging phenotype. This review summarizes current evidence and highlights ongoing clinical trials, underscoring a paradigm shift toward precision medicine and rational combination strategies. Collectively, these advances bring cautious optimism that metastatic PPGLs may soon become a more manageable chronic disease with improved survival and quality of life.

## Simple Summary

Metastatic pheochromocytomas and paragangliomas (PPGLs) are rare neuroendocrine neoplasms characterized by excess catecholamine production and secretion. This review summarizes current systemic therapy options and emerging strategies. Radiopharmaceuticals (^131^I-MIBG, ^177^Lu-DOTATATE) and cytotoxic regimens (CVD, temozolomide) remain cornerstone treatment options for disease control. Multi-targeted tyrosine kinase inhibitors (sunitinib, cabozantinib, axitinib) and the recently approved HIF-2α inhibitor belzutifan offer other targeted options, particularly for some PPGLs. Immune checkpoint inhibitors have modest activity, but combination approaches with TKIs or PARP inhibitors are currently under investigation. It is increasingly recognized that treatment for these tumors should be individualized based on tumor kinetics, genotype, and functional imaging phenotype, with multidisciplinary involvement and clinical trial participation being encouraged.

## 1. Introduction

Pheochromocytomas and paragangliomas (PPGLs) are rare neuroendocrine neoplasms, with an estimated incidence of approximately 0.6 per 100,000 individuals per year. Selected cohorts show survival differences by metastatic presentation and suggest that cytoreductive surgery may improve outcomes in carefully chosen patients, underscoring the need for effective systemic therapies [1].

PPGLs are characterized by the overproduction of catecholamines, and often present with symptoms that mimic around 30 other medical conditions [2]. Common clinical manifestations include a diverse range of symptoms/signs, such as palpitations, hyperhidrosis, headaches, and anxiety. The overlap between these presentations and those of more prevalent disorders complicates the diagnostic process for PPGLs [3]. Clinicians typically evaluate three key considerations: (i) elevated levels of free plasma or urinary metanephrines (ii) evidence of the adrenal gland or retroperitoneal masses, and (iii) the presence of germline mutations in a PPGL susceptibility gene or specific molecular markers associated with the disease [4]. With approximately 20 known genetic mutations implicated in this condition, treatment approaches must be tailored to the individual patient [5]. Furthermore, the variability in patient presentations necessitates an understanding of how different individuals respond to the various levels of hormone secretion.

Surgical intervention is typically the first-line treatment for PPGL, and while there is some debate regarding the optimal surgical technique and timing, it is generally regarded as a curative option [6]. However, certain metastatic PPGLs may be unresectable, necessitating alternative therapeutic strategies. The timeline for implementing these alternatives is contingent upon disease progression and tumor burden, as well as the presence of mass effect. Patients exhibiting slow progression and low tumor burden are often placed under surveillance with no immediate therapeutic intervention, unless the disease involves a critical area with limited anatomical space, wherein earlier treatment may be warranted. Conversely, those with slow-to-moderate progression but moderate-to-high tumor burden are generally treated using radionuclide therapies such as peptide receptor radionuclide therapy (PRRT) and I131-Metaiodobenzylguanidine (^131^I-MIBG) therapy. In cases of rapid disease progression and significant visceral tumor burden, chemotherapy regimens may be instituted. Should these treatment strategies prove ineffective, patients may then be considered for therapies such as tyrosine kinase inhibitors [7]. If these options also yield insufficient results, participating in clinical trials may be recommended as a viable means of exploring additional treatment avenues that could potentially be beneficial for the disease [8].

This review aims to evaluate various treatment modalities and recent advances in systemic therapies for metastatic PPGLs. We integrate post-approval evidence for the HIF-2α inhibitor belzutifan in advanced PPGLs and discuss practical positioning relative to TKIs, radiopharmaceuticals, and chemotherapy. We synthesize the growing prospective and real-world PRRT evidence that is specific to PPGLs. Finally, we include a genotype–phenotype–therapy correlation table to facilitate personalized care

## 2. Definition of Pheochromocytoma and Paraganglioma (PPGL)

Pheochromocytoma and paraganglioma (PPGL) are rare neuroendocrine tumors that arise from chromaffin cells of the adrenal medulla or from the neural crest progenitors located outside of the adrenal gland [9]. According to the WHO Classification of Endocrine and Neuroendocrine Tumors, paragangliomas are non-epithelial neuroendocrine neoplasms that generally produce catecholamines and secrete them into the bloodstream [10]. Approximately 80–85% of tumors are located in the adrenal medulla (pheochromocytomas), while 15–20% arise in extra-adrenal sites (paragangliomas) [2]. Tumors from the sympathetic chain (thorax, abdomen, pelvis) are usually catecholamine-producing, whereas those from parasympathetic tissues in the head and neck region are typically non-secreting, with only ~4% producing catecholamines [2].

Histologically, sustentacular cells (S-100/SOX10 positive) surround tumor nests and may provide supportive diagnostic information, especially in the distinction of primary from metastatic tumors [10]. The Ki-67 proliferation index can be used to assess proliferative activity, and if high, it is suggestive of a more aggressive tumor, often reflecting metastatic ones. The criterion for malignancy in PPGLs is the presence of metastases at sites where chromaffin tissue is normally absent: currently only in bones or lymph nodes [11].

## 3. Genetic Risk Factors

PPGLs are among the most heritable tumors: 30–40% harbor germline pathogenic variants overall, with pediatric cases showing ~70–80% germline prevalence [12]. The most common hereditary drivers include *SDHx* (*SDHA*/*SDHB*/*SDHC*/*SDHD*/*SDHAF2*), *VHL*, *RET*, *NF1*, *TMEM127*, and *MAX*, among others; *SDHx* accounts for ~20% of all PPGLs [13].

Most genetic mutations linked to PPGLs can be divided into three clusters (see Table 1): pseudohypoxia (Cluster I), kinase signaling (Cluster II), and Wnt-signaling (Cluster III) [14]. Cluster I encompasses *VHL*, *EPAS1*, and TCA-cycle genes (*SDHx, FH*, *MDH2*, *SLC25A11*, etc.) [15]. Genetic mutations related to Cluster II include *RET*, *NF1*, *TMEM127*, *MAX*, and *HRAS.* Cluster III includes *UBTF-MAML3* fusions and *CSFE1* mutation [16].

Metastatic propensity is gene-specific, rather than cluster-wide: *SDHB/A*, *HIF2A,* and *MAML3* (and to some degree, *FH*) confer high metastatic risk, whereas RET/NF1/TMEM127 are generally low-risk; MAX carries an intermediate risk (~15–20%) [17]. In children, presentation more often includes sustained hypertension, reflecting persistent catecholamine excess. Although research is limited, especially for PPGLs that belong to Cluster 3, it is thought that all current therapies can be potentially beneficial; however, they are not highly successful or mainly curative in PPGL patients of all cluster mutations [18]. The efficacy of treatment depends on more than the specific gene mutations, due to the individuality of the disease in each patient. Below is a table outlining the different clusters of mutations, along with their cluster characteristics and common gene mutations.

## 4. Local Therapeutic Approaches

### 4.1. Surgery

When PPGLs are localized, they can be surgically resected [19]. While surgery may be a potentially curative treatment for localized tumors, in metastatic disease, surgery is primarily palliative [7]. Despite this, a retrospective study with 113 patients showed an increase in the median overall survival from 36 to 148 months in patients with a metastatic disease undergoing debulking surgery [20]. Even if a tumor is localized, the size and location of the tumor can provide major difficulties in being able to effectively and safely resect the tumor. For adrenal tumors, unilateral adrenalectomy usually preserves adequate adrenal function; thus, lifelong steroid replacement is required only after bilateral adrenalectomy [21]. In hereditary/bilateral contexts, a partial adrenalectomy may reduce lifelong steroid dependence [22]. Despite these challenges, surgery is still one of the most effective ways to remove these tumors [23].

In preparation for surgery, alpha adrenoceptor blockers are initiated 7–14 days before for vasodilation and to decrease blood pressure, which can help regulate excessive hormone secretion [3]. Following alpha-blockade, beta blockers may be needed to help stabilize the patient’s cardiovascular system during surgery [4]. Calcium channel blockers are suitable adjuncts/alternatives in selected patients. Metyrosine, that is inhibiting catecholamine synthesis (50–80% reduction) [24], may be considered in patients with high catecholamine burden or suboptimal control.

### 4.2. Liver-Directed Therapies

Liver-directed therapies may be indicated in a subset of patients, particularly those with oligometastatic disease or a symptomatic lesion, with the intent to provide local control and palliation [25]. Liver tumors that are less than 2–3 cm can be more difficult to resect and those may be ablated [7]. Ablation is performed under ultrasound or CT guidance; device power and time are tailored (typical ranges ~60–150 W for ~3–10 min) [26,27,28].

A single center retrospective series reported different types of ablation of 123 metastases in 31 patients (24 PCC, 7 PPGL). These metastases were in various locations, including bone (51%), liver (44%), and other locations (5%). In this study, radiofrequency ablation (61%), cryoablation (33%) and percutaneous ethanol injection (6%) were used as treatments. The technical success was around 94%. A total of 80 of the metastases had imaging follow-ups, and among these, 86% achieved local control, which was equal for RFA and cryoablation. Local control was 94% in the liver (all RFAs), 88% bone cryoablation, and 74% for bone RFA. Pain control was achieved in 100% of the subjects [29].

An alternative to ablation is liver embolization. Transcatheter arterial embolization (TAE) is a type of liver embolization that is potentially used for decreasing free catecholamines. A few case studies have reported that TAE could perform control of hyper catecholamine secretion [30]. This procedure has been demonstrated as an acceptable bridge to elective surgical tumor resection [30]. Case reports describe rapid BP stabilization in catecholamine crisis after TAE, and in ruptured pheochromocytoma, tumor shrinkage enabled delayed elective surgery (e.g., reduction from 5.7 cm to ~1.0 cm at 6 months) [31]. While these data are promising, it is important to consider that further studies are needed to confirm the efficacy of TAE in reducing tumor size in ruptured pheochromocytomas.

## 5. Current Systemic Therapies for PPGL

### 5.1. Somatostatin Analogs

Neuroendocrine tumors tend to overexpress somatostatin receptors (SSTRs). In PPGL, SSTR expression is common at about 90% in metastatic disease, but varies by subtype [32]. In a cohort of 202 PPGLs patients, 50% were SSTR2 positive [33]. Another study observing 151 primary tumors found that over half of the tumors strongly expressed either SSTR2 or SSTR3 [34]. Patients with PPGLs can be treated with somatostatin analogs (SSAs), which can suppress hormone release and tumor cell proliferation [35], but high-level efficacy data with “cold” SSAs in PPGLs remain limited [32]. In practice, there are three main Somatostatin analogs (SSAs) used in treating PPGLs: octreotide, lanreotide, and pasireotide [36]. Antitumor efficacy and approval is established in GEP-NETs for Octreotide and Lanreotide (PROMID and Clarinet trial) [37,38], but there is limited PPGL- specific data. A dedicated Phase II in PPGLs (LAMPARA) is ongoing (see Table 2) [39].

Pasireotide is the newest SSA and it targets somatostatin receptors SSTR1, SSTR2, SSTR3, and SSTR5 [40]. Additionally, Pasireotide has a 40-fold higher affinity for SSTR5, 30-fold higher for SSTR1, and 5-fold higher for SSTR3, compared to octreotide [41].

Pasireotide is approved for targeting acromegaly and Cushing’s, not NET/PPGL; in a Phase III study of refractory carcinoid syndrome, pasireotide did not outperform octreotide for symptom control, with exploratory signals for longer PFS [40].

### 5.2. Peptide Receptor Radionuclide Therapy PRRT

Peptide receptor radionuclide therapy (PRRT), with ^177^Lu-DOTATATE, is used to treat somatostatin-receptor-positive PPGLs and has shown activity with acceptable toxicity in multiple cohorts [42,43]. A nationwide retrospective study in Denmark, which included patients who had been diagnosed with PPGLs and had received PRRT over a 15 year period, showed that patients with a germline mutation, including SDHB, who received PRRT had better overall survival than patients without germline mutations (*p* = 0.041) [44]. The median follow-up time for this study was 31 months, and it showed an overall survival benefit for those with germline mutations. Patients with SDHB had a PFS of about 18 months, which was not achieved by patients with other mutations like SDHD or RET. Importantly, it should be noted that ^177^Lu-DOTATATE is associated with transient significant endocrine fluctuations and catecholamine surges, typically 24–48 h after administration, that are subsequently normalized. These potentially persistent effects are seen at a greater rate in more powerful agents, such as alpha particle PRRT [45]. A study performed in Stanford investigated six patients (one woman and five men) who expressed PPGL, four paraganglioma and two pheochromocytoma, using four 7.4 GBq cycles: PFS was 83%, 58%, and 39% at 11, 15, and 18 months, ORR was 40% at post-treatment assessment, disease control was 80% at 11 months, and mainly transient grade-3 cytopenias were without grade-3 hepatic/renal toxicity [46].

Prospectively, Lin et al. [47] conducted a phase-II Simon two-stage trial (four 7.4 GBq cycles; 36 evaluable) and reported a 6 month PFS rate of 86%, median PFS 20 months, and deeper responses over time (partial responses rising from 14% at end-of-treatment to 28% at follow-up). Outcomes differed by genotype: sporadic tumors had longer PFS (24.3 months) than *SDHx* tumors (12.9 months). Treatment was generally well tolerated but included 18% grade ≥ 3 catecholamine-release events.

In a case study with five patients, it was observed that when alpha/beta-blockers and calcium channel blockers were used to treat patients before their injections, there were no infusion reactions or catecholamine crises [48]. Overall, compared with “cold” SSAs (octreotide/lanreotide), PRRT in appropriately selected, SSTR-avid PPGLs provides higher rates of radiographic response and disease stabilization across a series, with an evolving but supportive safety profile [7,42].

### 5.3. Radioligand Therapy (MIBG)

PPGL frequently express the norepinephrine (NE) reuptake transporters on their cell surface. Meta-iodobenzylguinidine (MIBG) are substrates for these norepinephrine reuptake transporters that compete for binding sites and disrupt the NE reuptake mechanism; hence, reduction in the circulation of catecholamines. Highly specific MIBGs are labeled with radioactive iodine (^131^I-MIBG) and are used for specific targeted therapy [49]. In the pivotal phase II trial, patients received a dosimetric planning dose, followed by therapeutic doses of ~18.5 GBq (500 mCi) ~90 days apart. Among 64 evaluable patients, 92% achieved a partial response or stable disease within 12 months; 25% of all treated patients met the primary endpoint of a durable ≥50% reduction in antihypertensive medications. The median OS was ~36.7 months. Common AEs were myelosuppression, nausea, and fatigue, and no drug-related acute hypertensive crises occurred during/after infusion [50]. Overall, most patients treated with this drug achieved a decrease in tumor size, and lessened disease and symptoms as well. The findings showed a sustained control of catecholamine-associated hypertension in a quarter of the patients [51]. Not all PPGLs are sufficiently MIBG-avid for therapy, so avidity testing is essential [52]. Overall, for MIBG-avid disease, HSA ^131^I-MIBG achieves high rates of tumor control and biochemical/pressoric improvement in pretreated PPGL populations, often with a toxicity profile that is more manageable than multi-agent cytotoxic chemotherapy [53].

When both somatostatin SSTR and MIBG imaging demonstrate a therapeutically relevant uptake, the treatment selection should prioritize (1) dominant avidity and lesion-level heterogeneity, favoring the modality with stronger and more homogeneous uptake across sites; (2) bone marrow reserve and prior hematologic exposure, as both PRRT and ^131^I-MIBG can induce cytopenias; (3) renal function and the feasibility of amino acid nephroprotection with PRRT; (4) secretory status, given the transient catecholamine surges reported after PRRT that necessitate an optimal α/β blockade; (5) logistical and institutional radiation safety considerations; and (6) prior therapies and cumulative toxicity, to minimize additional marrow suppression when possible.

Head-to-head comparative data between PRRT (e.g., ^177^Lu-DOTATATE) and ^131^I-MIBG are lacking. No randomized trials directly compare these modalities, and the available evidence derives from small retrospective or nonrandomized series with inherent selection bias. In the largest PPGL cohort, PRRT was associated with longer overall and progression-free survival and higher response rates than ^131^I-MIBG, though results were confounded by referral and imaging bias [54]. A comparison reported a trend toward longer progression-free survival with ^177^Lu-DOTATATE versus ^131^I-MIBG (median of 29 vs. 18.5 months), but the difference was not statistically significant [55]. Other single-center series have similarly suggested improved outcomes with PRRT in select dual-avid PPGL cases, though these findings are limited by small sample sizes and nonuniform eligibility criteria [56]. Overall, direct comparative evidence remains absent, and existing data only suggest potential advantages for PRRT in subsets of dual-avid patients. Accordingly, therapy selection should remain individualized, and guided by imaging dominance, marrow reserve, renal and secretory status, prior therapies, and institutional expertise, while recognizing that PRRT and ^131^I-MIBG have distinct regulatory indications and toxicity profiles that complicate a direct comparison [57].

### 5.4. Tyrosine Kinase Inhibitors

Tyrosine kinases are critical mediators of oncogenic signaling pathways that regulate cancer cell proliferation, survival, and metastasis. Tyrosine kinase inhibitors (TKIs) represent a class of targeted agents that are designed to block aberrant kinase activity, thereby disrupting tumor growth and progression [58]. Patients with PPGLs have higher rates of angiogenesis and overexpression of growth factor receptors, such as vascular endothelial growth factor (VEGF) and VEGF receptors (VEGFR) [8]. This is especially relevant in patients with Cluster 1 (pseudohypoxia) tumors, such as *SDHx/VHL/EPAS1*, where hypoxia signaling promotes angiogenesis. TKIs are thought to be effective in treating PPGLs in part due to their ability to inhibit angiogenesis [18].

TKIs are typically used to treat unresectable tumors and metastatic PPGLs. A meta-analysis demonstrated an ORR of 32%, a DCR 86%, and a median PFS of 8.9 months [59]. Common adverse effects of TKIs include hypertension, fatigue, diarrhea, and PPES (palmar-plantar erythrodysesthesia syndrome). While roughly one in seven patients do discontinue TKIs due to toxicity [59], it is common for patients to reduce their dosage to better tolerate adverse effects.

The most prevalent TKIs are sunitinib, axitinib, and cabozantinib (see Table 3) [60]. In progressive metastatic PPGLs specifically, the FIRSTMAPP randomized, double-blind phase II trial, met its primary endpoint with 12 month PFS 36% in the sunitinib group versus 19% in the placebo group [61,62]. The overall response rate to sunitinib for metastatic PPGLs is generally low; however, a small series suggests that SDHx-mutated tumors may respond to anti-angiogenic TKIs, and RET-altered pheochromocytomas have responded to selective RET inhibitors (e.g., selpercatinib), although robust predictive evidence for greater benefit with multi-target TKIs by genotype remains limited [63]. Retrospective data also note blood-pressure improvement in some patients on sunitinib [62].

On the other hand, a Phase 2 trial with axitinib (NCT01967576) indicated partial responses in three out of nine patients and stable disease in five out of nine [64]. For cabozantinib, the single-arm phase II NATALIE trial (NCT02302833) in progressive metastatic PPGLs showed an ORR of 25% and a median PFS of 16.6 months [65]. Responses were observed regardless of SDHB mutation status, indicating that cabozantinib is effective across both hereditary (SDHB-driven) and sporadic cases.

Additionally, anlotinib, a multi-target VEGFR/FGFR/PDGFR TKI (12 mg daily, 2 weeks on/1 week off), has shown encouraging real-world activity in advanced PPGLs, with ORR 44%, DCR 96%, and median PFS 25 months [66]; prospective phase II trials in PPGLs are ongoing (NCT04860700, NCT05133349).

Overall, while the ORR to single-agent TKIs is modest, clinically meaningful disease control is common, and dose modifications are frequently used to manage toxicity [59].

**Table 3:** Tyrosine kinase inhibitors.

Drug	Dose	Target	Patient Population Studied	Efficacy	Side Effects
Sunitinib [61]	37.5 mg qd (FIRSTMAPPP), 50 mg qd also used.	VEGFR1–3, PDGFRα/β, KIT, FLT3, RET	Progressive/metastatic PPGLs (sporadic and germline)	FIRSTMAPPP (phase II RCT): 12 months PFS 36% vs. 19% (sunitinib vs. placebo). SUTNET (phase II, non-randomized): median PFS 14.1 months.	HTN, fatigue/asthenia, diarrhea. Grade ≥3 in FIRSTMAPPP: asthenia 18%, HTN 13%.
Cabozantinib [65]	60 mg qd.	VEGFR2, MET, AXL, RET (multi-target)	Unresectable, progressive metastatic PPGL	NATALIE (phase II, single-arm): ORR 25%; median PFS 16.6 months; median OS 24.9 months.	HTN, hand-foot syndrome, dysgeusia, fatigue.
Axitinib [64]	5 mg bid.	VEGFR1–3 (selective)	Progressive PPGL	Phase II (prelim.): PR 3/9, SD 5/9 (DCR ~89%).	HTN, fatigue; mucositis; Diarrhea.

### 5.5. Chemotherapy (See Table 4)

#### 5.5.1. Cyclophosphamide–Vincristine–Dacarbazine (CVD) Regimen

The most common forms of chemotherapy include cyclophosphamide, vincristine, and dacarbazine (CVD) and temozolomide (TMZ) to treat advanced or unresectable PPGL. In one of the earliest CVD reports, Keiser et al. described three patients with rapidly progressive malignant pheochromocytoma who experienced marked blood pressure reductions and clinical improvement within the first cycles of treatment [67].

**Table 4:** Cytotoxic chemotherapy.

Drug/Regimen	Dose	Mechanism/Target	Study Group	ORR (in %), PFS (in Months), OS (in Months)	Side Effects
CVD (cyclophosphamide–vincristine–dacarbazine)	Cyclophosphamide 750 mg/m^2^ D1; vincristine 1.4 mg/m^2^ (max 2 mg) D1; dacarbazine 600 mg/m^2^ D1–2; q21–28 days (Averbuch protocol).	Cytotoxic combo: alkylator (cyclophosphamide), vinca (vincristine), alkylator (dacarbazine).	Metastatic/unresectable PPGL.	Small series show ORR 55–57%; meta-analysis (4 studies) CR 4%/PR 37%/SD 14%; 1 year PFS 45% in one cohort; OS inconsistently reported [68,69,70,71]	Mostly grade 1–2 myelosuppression, neuropathy, GI upset; rare hypertensive crises reported (optimize α/β-blockade).
Temozolomide (TMZ)	150–200 mg/m^2^ PO daily D1–5 q28 days (monotherapy); in Kulke trial: TMZ 150 mg/m^2^ + thalidomide 100 mg daily.	Oral alkylating agent; activity linked to MGMT promoter hypermethylation (frequent in SDHB PPGL).	Metastatic PPGLs (retrospective series); mixed NET phase II included small PCC cohort.	Across PPGL series: PR 33–40%; median PFS 13.3 months–~26 months; 3 years OS 58% in one cohort. (NET phase II overall ORR 25%, median response 13.5 mo; PCC subset included) [72 73]	Generally well tolerated; lymphopenia/myelosuppression, fatigue, nausea.

In a larger non-randomized, single arm trial by Averbuch et al. (n = 14), those diagnosed with metastatic PPGLs showed an overall response rate (ORR) of 57% and the trial showed complete or partial biochemical responses in 79% of patients, supporting activity of CVD in metastatic disease [68].

A 22 year follow-up study by Huang et al. (n = 18) reported CR 11% and PR 44% (ORR 55%), with consistent symptom and blood-pressure improvement and acceptable tolerability [69].

A systematic review and meta-analysis, pooling four studies (50 patients), estimated complete responses at 4%, partial responses at 37%, stable disease at 14% for tumor volume, and partial hormonal response at 40% (catecholamines) where reported, suggesting that CVD can palliate and shrink a tumor in a subset [70].

#### 5.5.2. Temozolomide

Temozolomide (TMZ) is an oral alkylating chemotherapeutic again. A Phase II study was performed to determine the efficacy of TMZ in patients with metastatic NETs (n = 29). The radiologic response rate was 25% overall, with responses by subtype being 45% in pancreatic NETs, 33% in pheochromocytoma, and 7% in midgut NET; the median response duration was 13.5 months, 1 year OS was 79%, and 2 year OS was 61% [72]. Overall, the study showed that TMZ was an active treatment in pheochromocytoma. In metastatic PPGLs specifically, Hadoux et al. (n = 15) reported PR at 33%, all among SDHB-mutated tumors; median PFS at 13.3 months; and longer PFS for SDHB-mutated vs. non-mutated cases (19.7 vs. 2.9 months). SDHB status correlated with the MGMT promoter hypermethylation/low MGMT expression: a plausible mechanism for TMZ sensitivity [73].

A more recent retrospective series (Perez 2022, n = 19) found PR at 40% (6/15 evaluable), SD at 27%, PD at 33%, a median PFS of 2.2 years, and 3 year OS at 58%; in that cohort, outcomes did not favor SDHx carriers over noncarriers, highlighting heterogeneity across studies [74].

Pediatric data are limited, but a case report of a 12-year-old girl with SDHB-related metastatic PGL treated with TMZ after surgery documented clinical improvement and good tolerability, suggesting that TMZ can be considered in carefully selected children [75].

### 5.6. Immunotherapy in Advanced PPGL

In the first prospective trial of the PD-1 blockade, pembrolizumab monotherapy showed modest activity: only 1 of 11 patients (9%) achieved a partial response, but 40% of patients were progression-free at 27 weeks and the clinical benefit rate (including stable disease) reached about 72% [76]. Notably, treatment was tolerable with an acceptable safety profile. These results indicate that single-agent immunotherapy can stabilize disease in a subset of patients, though deep responses are rare. Dual checkpoint blockade is now under exploration; for example, a phase II trial combining nivolumab (anti–PD-1) with ipilimumab (anti–CTLA-4) in metastatic PPGLs is ongoing [77]. Early anecdotal reports also hint that combining immunotherapy with other agents may enhance efficacy. For instance, one case report described a dramatic and durable tumor regression when nivolumab was given together with the multi-kinase inhibitor cabozantinib, after multiple prior treatment failures [78].

Multiple datasets delineate a cluster-dependent immune contexture in PPGL. PD-L1 expression is generally low and tends to be lowest in pseudohypoxia-cluster tumors (*SDHx*, *VHL*, *EPAS1*), relative to kinase-signaling and sporadic cases, aligning with the modest activity seen in single-agent PD-1/PD-L1 blockade [79]. CD8^+^ T-cell infiltration shows a similar pattern (reduced in pseudohypoxic tumors) [80]; norepinephrine-secreting tumors also display lower PD-L1/CD8 than epinephrine-secreting diseases [81]. An exception is the MAML3-related subset, where PD-L1 positivity and a less immunosuppressive microenvironment have been reported [13]. This biology tempers expectations for a single-agent checkpoint blockade but does not preclude benefit in a subset of patients.

Future studies are focusing on combination approaches: pairing checkpoint inhibitors with targeted therapies or novel immunomodulators to improve response rates in this historically immunoresistant disease.

## 6. Novel Targeted Therapies: HIF-2α Inhibition and Other Strategies

A major advance in 2025 was the introduction of belzutifan, a selective inhibitor of hypoxia-inducible factor-2α (HIF-2α). In May 2025, the FDA approved belzutifan for adult and pediatric patients ≥12 y with locally advanced, unresectable, or metastatic PPGLs [82]. This approval was based on the LITESPARK-015 trial (n = 72 open label), in which belzutifan achieved a 26% objective response rate with a median duration of response (DOR) of 20.4 months; disease control was 85%, median PFS was 22.3 months, and 24 month OS was 76%. Among the 60 patients on baseline antihypertensives, 32% achieved a ≥50% sustained reduction in at least one medication for ≥6 months. Safety was manageable and consistent with prior experience; anemia was the most common grade ≥3 event (22%) [83,84]. Belzutifan targets the HIF-2α transcription factor, a known oncogenic driver in pseudohypoxic PPGLs [58]. This mechanism may be particularly relevant to SDHB-related tumors and other Cluster 1 PPGLs (e.g., VHL- and EPAS1-mutated), which are driven by dysregulated hypoxia signaling [58]. Indeed, HIF-2α inhibitors were long theorized as being promising agents for these patients, and belzutifan’s clinical success confirms that targeting the HIF pathway can yield meaningful tumor responses in advanced PPGL.

Going forward, belzutifan may provide a much-needed targeted option: it is an oral agent that can induce partial remissions and durable disease control, and it is being integrated into treatment algorithms, particularly for patients with Cluster 1 mutations.

Beyond HIF-2α, other molecular targets are under investigation. PARP inhibitors are one example: SDHB-mutant PPGLs accumulate oncometabolites (succinate) that may confer vulnerability to the PARP blockade [85]. Preclinical studies showed that adding the PARP inhibitor olaparib can amplify temozolomide’s cytotoxicity in SDHB-deficient models [58]. This has led to early-phase trials combining temozolomide with PARP inhibitors (e.g., talazoparib or veliparib) in metastatic PPGL. Another emerging strategy is exploiting the metabolic vulnerabilities of SDHB-related tumors. For instance, research is ongoing into targeting the unique metabolic pathways and redox state of SDHB-null cells (such as through oxidative stress induction or Krebs cycle enzyme modulation) [86]. While these approaches remain investigational, with belzutifan’s success, there is optimism that novel targeted agents, from HIF-2α antagonists to PARP inhibitors and beyond, can meaningfully improve outcomes.

## 7. Enhanced Treatment Sequencing and Future Directions

As of 2025, many active clinical trials are actively investigating treatments for PPGLs, many of which involve innovative combinations, such as cancer vaccines [87,88] with checkpoint inhibitors, novel immune drugs with PD-1 inhibitors, and multi-drug regimens incorporating PARP inhibitors, targeted agents, and chemotherapy [89,90]. The preliminary signals from these studies are encouraging, though full published data are awaited.

The expanding arsenal of systemic therapies has made treatment sequencing an increasingly nuanced aspect of PPGL care. An individualized approach is crucial, considering the tumor growth rate, disease burden, functional status, and tumor genotype or avidity (see Table 5).

The optimal sequencing is not yet defined by guidelines, but multidisciplinary expert consensus emphasizes personalization [18]. Factors such as germline mutation status, tumor differentiation, and dominant imaging phenotype (MIBG-avid vs. SSTR-avid vs. FDG-avid) [91] guide therapy selection at each juncture.

Emerging biomarkers may further refine sequencing by indicating who is benefiting from a given treatment [25]. In all cases, the principle of maximizing disease control while minimizing cumulative toxicity prevails [51]. This often means using the least aggressive effective therapy first and escalating as needed, like starting with a radioligand or targeted agent before switching to multi-agent chemotherapy or experimental combinations. It also means enrolling patients in clinical trials whenever possible, so that they can access novel therapies.

As ongoing trials broaden our options, three real-world constraints warrant emphasis. First, therapeutic resistance: angiogenic escape with multi-target TKIs is common, and on-target resistance to HIF-2α inhibitors (e.g., EPAS1 G323E gatekeeper variants and the compensatory HIF-1 signaling described in RCC) may analogously limit durability in PPGLs [59,92]. Second, patient selection: lesion-level heterogeneity of SSTR/MIBG expression and the risk of catecholamine surges after PRRT require pre-treatment α/β-blockade, endocrine monitoring, and multidisciplinary planning [33,45]. Third, access and logistics: availability and reimbursement vary by region, and high-specific-activity ^131^I-MIBG production was discontinued in 2023 [93], which can influence sequencing choices. Priorities include trials integrating genotypes (e.g., *SDHB*, *VHL/EPAS1*), imaging phenotypes, and immune contextures, while capturing affordability and capacity constraints as new agents (e.g., belzutifan) move earlier in care.

## 8. Conclusions

Metastatic or unresectable pheochromocytomas and paragangliomas are uncommon but clinically challenging. The systemic armamentarium now spans radiopharmaceuticals (^131^I-MIBG and ^177^Lu-DOTATATE PRRT), cytotoxic chemotherapy (CVD and temozolomide), anti-angiogenic TKIs (e.g., sunitinib, cabozantinib, and axitinib), and the recently approved HIF-2α inhibitor belzutifan, with a single-agent immune checkpoint blockade showing modest activity. Optimal use of these modalities is individualized to tumor kinetics, germline/somatic genotype, and functional imaging phenotype (MIBG- or SSTR-avid disease), with attention to safety and local availability.

Looking ahead, priorities include biomarker-driven sequencing and rational combinations to overcome resistance and heterogeneity (e.g., PARP inhibitor–temozolomide strategies in *SDHB* disease, TKI–ICI combinations), as well as a pragmatic assessment of access, toxicity, and patient-reported outcomes. Prospective, genotype- and imaging-embedded trials remain essential to define comparative effectiveness and translate molecular insights into durable disease control and an improved quality of life for patients with PPGLs.

## Figures and Tables

**Table 1:** Cluster characteristics and common gene mutations [14,16].

Cluster #	Cluster Characteristic	Common Gene Mutations
Cluster I	Pseudohypoxia	HIF-pathway: *VHL*, *EPAS1 (HIF2A)*, *EGLN1 (PHD1)*, *EGLN2 (PHD2)* TCA-cycle/SDH complex: *SDHA*, *SDHB*, *SDHC*, *SDHD*, *SDHAF2*, *FH*, *MDH2*, *SLC25A11*, *DLST*
Cluster II	Kinase Signaling	*RET*, *NF1*, *TMEM127*, *MAX*, *HRAS*
Cluster III	Wnt-Signaling	*UBTF*–*MAML3* fusions (*MAML3*), *CSDE1*

# Number.

**Table 2:** Somatostatin analogs.

Drug	Usual Dose and Route	Primary SSTR Target(s)	Population/Key Results	PPGL-Specific Notes	Common AEs	Key Trial(s) and n
Octreotide LAR	20–30 mg IM q28 days	SSTR2 > SSTR5	Metastatic midgut NETs (PROMID): TTP 14.3 vs. 6.0 months vs. placebo; HR 0.34; SD at 6 months 66.7% vs. 37.2%.	“Cold” SSA use in mPPGL mainly for biochemical control/stabilization; antiproliferative data insufficient, guidelines cannot recommend for/against [7].	Diarrhea, biliary sludge/cholelithiasis, hyperglycemia, bradycardia.	* PROMID (JCO 2009) n = 85; [37]
Lanreotide (Autogel/Depot)	120 mg SC q28 days	SSTR2	Nonfunctioning G1–2 GEP-NETs (CLARINET): median PFS not reached vs. 18 months; HR 0.47; 24 months PFS 65% vs. 33%.	Not FDA-approved for PPGL. Reasonable for symptom control/stabilization when SSTR-avid [7].	Diarrhea, abdominal pain, cholelithiasis; injection-site reactions.	* CLARINET (NEJM 2014) n = 204; [38]
Pasireotide LAR	40–60 mg IM q28 days (trial used 60 mg)	SSTR5 > SSTR1/3; also SSTR2	Refractory carcinoid syndrome (NETs): symptom control similar to high-dose octreotide LAR; exploratory PFS 11.8 vs. 6.8 months, HR 0.46 (post hoc).	Not approved for NET/PPGLs (approved for acromegaly/Cushing’s). PPGL data limited to preclinical/very small series. Investigational use [7].	Hyperglycemia (notably higher than octreotide), GI upset, cholelithiasis.	* Wolin et al. Phase III (2015) n = 110; [40]
Lanreotide–LAMPARA (Phase II)	120 mg SC q28 days	SSTR2	Advanced SSTR-positive PPGL; single-arm (~40 planned). Primary endpoint: change in tumor growth rate on-treatment vs. pre-enrollment; secondary: PFS, OS, ORR. Results pending.	First prospective PPGL trial of a cold SSA; aims to clarify antiproliferative signal in PPGLs [39].	As per lanreotide label (above).	NCT03946527.

* Not a PPGL trial.

**Table 5:** Molecular clusters, imaging phenotype and therapeutic implications in PPGLs.

Cluster/Gene(s)	Functional Imaging: Expected Uptake and First-Line Tracer	Therapies with Strongest Signal
Pseudohypoxia (1A) *SDHB/SDHx*	Phenotype: highly FDG-avid, SSTR-positive; MIBG variable/low First-line: SSTR PET/CT	Cyclophosphamide–Vincristine–Dacarbazine (CVD);Temozolomide (SDHB/MGMT-linked sensitivity); PRRT when SSTR-avid disease; PARP + TMZ (investigational)
Pseudohypoxia (1B) *VHL/EPAS1*	Phenotype: SSTR-positive; FDOPA avid (esp. adrenal PHEO); MIBG variable First-line: FDOPA PET/CT *or* SSTR PET/CT	Belzutifan (HIF-2α inhibitor); PRRT if SSTR-avid disease
Kinase signaling (2) *RET*, *NF1*, *TMEM127*, *MAX*, *HRAS*	Phenotype: SSTR-positive; FDOPA avid; MIBG variable. First-line: FDOPA PET/CT *or* SSTR PET/CT	TKIs (sunitinib, cabozantinib, axitinib); PRRT if SSTR-avid disease
Wnt/other (e.g., *MAML3)*	Phenotype: Variable First-line:SSTR PET/CT as pragmatic default.	Consider ICI in trials (PD-L1 subset); PRRT if SSTR-avid disease
MIBG avid disease (any genotype)	Phenotype: MIBG-avid First-line: ^123^I-MIBG SPECT/CT to document ^131^I-MIBG eligibility	^131^I-MIBG (availability/logistics noted in text)
SSTR avid disease (any genotype)	Phenotype: SSTR-positive; MIBG variable/negative. First-line: SSTR PET/CT	PRRT represents a core indication for SSTR-avid disease, demonstrating genotype-agnostic utility.

Note:  This summary does not constitute formal treatment guidelines. Therapeutic selection for PPGLs should be individualized based on disease growth kinetics, tumor distribution, SSTR and MIBG uptake patterns, biochemical secretory profile, prior systemic and radioligand therapies, bone marrow and renal reserve, and multidisciplinary assessment. The proposed framework is intended for academic and hypothesis-generating purposes only and should not substitute for guideline-based or patient-specific clinical decision-making.

## Data Availability

No new data were created or analyzed in this study. Data sharing is not applicable to this article.

## Abbreviations

The following abbreviations are used in this manuscript:

^131^I	Iodine-131
^177^Lu	Lutetium-177
AE(s)	Adverse event(s)
AXL	AXL receptor tyrosine kinase
bid	Twice daily
CVD	Cyclophosphamide–Vincristine–Dacarbazine
D1, D1–5	Day 1; Days 1 through 5
DOTATATE	DOTA-Tyr3-octreotate (Lu-177 DOTATATE)
FLR/FLT3	Fms-like tyrosine kinase 3 (FLT3)
GI	Gastrointestinal
GEP-NET	Gastroenteropancreatic neuroendocrine tumor
HFS	Hand-foot syndrome
HIF-2α	Hypoxia-inducible factor 2 alpha
HR	Hazard ratio
HTN	Hypertension
IM	Intramuscular
IRB	Institutional Review Board
KIT	KIT proto-oncogene receptor tyrosine kinase (CD117)
LAR	Long-acting release (e.g., octreotide LAR)
mg/m^2^	Milligrams per square meter
MGMT	O6-methylguanine-DNA methyltransferase
MIBG	Meta-iodobenzylguanidine
mo	Months
MET	MET proto-oncogene receptor tyrosine kinase
NET	Neuroendocrine tumor
NIH	National Institutes of Health
ORR	Objective response rate
OS	Overall survival
PDGFR	Platelet-derived growth factor receptor
PFS	Progression-free survival
PO	By mouth (per os)
PPGL	Pheochromocytoma and Paraganglioma
PR	Partial response
PRRT	Peptide receptor radionuclide therapy
qd	Once daily
q21–28d/q28d	Every 21–28 days/every 28 days
RET	RET proto-oncogene receptor tyrosine kinase
SC	Subcutaneous
SD	Stable disease
SDHB	Succinate dehydrogenase complex iron sulfur subunit B
SI-NET	Small-intestinal neuroendocrine tumor
SSTR	Somatostatin receptor
SSA	Somatostatin analog
TKI	Tyrosine kinase inhibitor
TMZ	Temozolomide
TTP	Time to progression
VEGFR	Vascular endothelial growth factor receptor
α/β-blockade	Alpha/Beta-adrenergic blockade
